# Factors Influence Dementia Treatment‐Seeking Behavior among Asian Indian Americans

**DOI:** 10.1002/alz70858_107198

**Published:** 2025-12-25

**Authors:** Anju Wadhawan

**Affiliations:** ^1^ Rutgers, The State University of New Jersey, Newark, NJ, USA

## Abstract

**Background:**

The Asian Indian American community represents the largest immigrant subgroup in the United States (US Census Bureau, 2020), with dementia prevalence projected to rise by 113% by 2040 (Alzheimer's Association, 2023). This escalating trend introduces significant caregiving challenges, including financial and emotional burdens, with unpaid caregiving estimated at $339.5 billion annually. Despite these challenges, Asian Americans remain the least likely racial group to receive dementia diagnostic services, revealing systemic healthcare disparities (Tsoy et al., 2021). Delayed treatment‐seeking is prevalent and influenced by stigma, cultural norms, and restricted healthcare access (Mayeda et al., 2017). Female caregivers often face compounded barriers, including economic hardship and role overload, further diminishing their quality of life (Chiari et al., 2020; Ng et al., 2021).

**Method:**

This literature review, guided by the Sociocultural Health Belief Model (Sayegh, & Knight, 2013) and Transcultural Nursing Theory (2002), examines factors influencing dementia treatment‐seeking behavior among Asian Indian American caregivers, focusing on three key domains: knowledge and awareness, health beliefs, and sociocultural factors.

**Result:**

Limited awareness about dementia and misconceptions, such as attributing cognitive decline to aging or spiritual causes, significantly delay treatment‐seeking (Casado et al., 2018; Jang et al., 2018). Cultural stigma, traditional caregiving norms, and reliance on familial support impede timely intervention, while acculturation and education levels influence healthcare utilization (Parial et al., 2023; Subramaniam et al., 2018). Health beliefs further complicate the issue, with caregivers often postponing medical help due to cultural stigmas, distrust in healthcare systems, or preferences for alternative treatments (Nguyen et al., 2022). Sociocultural factors, including stigma, acculturation, and systemic barriers, exacerbate the challenges in accessing care (Hossain & Khan, 2019; Tang et al., 2023).

**Conclusion:**

This review synthesizes findings to address the unique challenges faced by Asian Indian American caregivers. It highlights the urgent need for culturally sensitive interventions, health education, and policy reforms to facilitate early detection and treatment access. Overcoming these barriers is critical to improving health outcomes for individuals with dementia and their caregivers within the Asian Indian American community.

